# Establishment of an ELISA Based on a Recombinant Antigenic Protein Containing Multiple Prominent Epitopes for Detection of African Swine Fever Virus Antibodies

**DOI:** 10.3390/microorganisms12050943

**Published:** 2024-05-07

**Authors:** Dossêh Jean Apôtre Afayibo, Zhonghui Zhang, Hualin Sun, Jingsheng Fu, Yaru Zhao, Tharheer Oluwashola Amuda, Mengli Wu, Junzheng Du, Guiquan Guan, Qingli Niu, Jifei Yang, Hong Yin

**Affiliations:** 1 State Key Laboratory for Animal Disease Control and Prevention, African Swine Fever Regional Laboratory of China (Lanzhou), Lanzhou Veterinary Research Institute, Chinese Academy of Agricultural Sciences, Xujiaping 1, Lanzhou 730046, China; jeanafayibo@gmail.com (D.J.A.A.); dujunzheng@caas.cn (J.D.);; 2 Jiangsu Co-Innovation Center for the Prevention and Control of Important Animal Infectious Disease and Zoonosis, Yangzhou University, Yangzhou 225009, China

**Keywords:** African swine fever virus, indirect ELISA, B-cell epitopes, pig, endemic

## Abstract

African swine fever virus (ASFV) poses a significant threat to the global pig industry, necessitating accurate and efficient diagnostic methods for its infection. Previous studies have often focused on a limited number of epitopes from a few proteins for detecting antibodies against ASFV. Therefore, the current study aimed to use multiple B-cell epitopes in developing an indirect Enzyme-Linked Immunosorbent Assay (ELISA) for enhanced detection of ASFV antibodies. For the expression of recombinant protein, k3 derived from 27 multiple peptides of 11 ASFV proteins, such as p72, pA104R, pB602L, p12, p14.5, p49, pE248R, p30, p54, pp62, and pp220, was used. To confirm the expression of the recombinant protein, we used the Western blotting analysis. The purified recombinant K3 protein served as the antigen in our study, and we employed the indirect ELISA technique to detect anti-ASFV antibodies. The present finding showed that there was no cross-reactivity with antibodies targeting Foot-and-mouth disease virus (FMDV), Porcine circovirus type 2 (PCV2), Pseudorabies virus (PRV), Porcine reproductive and respiratory syndrome virus (PRRSV), and Classical swine fever virus (CSFV). Moreover, the current finding was sensitive enough to find anti-ASFV in serum samples that had been diluted up to 32 times. The test (k3-iELISA) showed diagnostic specificity and sensitivity of 98.41% and 97.40%, respectively. Moreover, during the present investigation, we compared the Ingenasa kit and the k3-iELISA to test clinical pig serum, and the results revealed that there was 99.00% agreement between the two tests, showing good detection capability of the k3-iELISA method. Hence, the current finding showed that the ELISA kit we developed can be used for the rapid detection of ASFV antibodies and used as an alternative during serological investigation of ASF in endemic areas.

## 1. Introduction

African swine fever (ASF) is a disease characterized by high mortality and morbidity rates among domestic and wild pig populations [1]. The disease is instigated by a DNA virus known as the African swine fever virus (ASFV), which has emerged in numerous countries worldwide, resulting in substantial losses within the pig production chain [2]. Furthermore, highly virulent strains of the virus can lead to acute hemorrhagic fever, frequently resulting in nearly 100% mortality rates. The disease manifests in acute, peracute, and chronic forms based on isolate virulence [3].

The genome of ASFV encodes a broad array of over 200 structural and non-structural proteins [2]. Among these proteins, p12, p17, p30, p54, p72, and CD2v are involved in viral replication, evading the immune system, and spreading the pathogen. For instance, p30 operates during the virus’s initial infection stage, while p54 aids in viral attachment and entry. The p72 protein is crucial for virion structure, ensuring effective immune response and stable antigens for serological diagnosis. Polyproteins pp62 and pp220 are essential for assembling virions and initiating viral infection. Encoded by the *E120R* gene, p14.5 is a significant protein synthesized late in viral infection, assisting in transporting virions from viral factories to the plasma membrane. Moreover, the inner envelope-based pE248R, with intramolecular disulfide bonds, is vital for virus infectivity, cell entry, and membrane fusion [1,4,5]. These antigenic proteins play a pivotal role in developing serological diagnostic tools for disease control and eradication, especially since a vaccine is not yet available following ASF outbreaks. Aligning ASF diagnostic techniques with local disease dynamics and human behavior is critical for effectively managing the disease [6].

The assessment of ASFV at different degrees of virulence, including high, moderate, and low levels in pigs, encompasses various detection methods developed in recent years. These methods include Polymerase Chain Reaction (PCR), Quantitative PCR (qPCR), Multiplex PCR, Enzyme-Linked Immunosorbent Assay (ELISA), Immunohistochemistry (IHC), Loop-Mediated Isothermal Amplification (LAMP), and Next-Generation Sequencing (NGS) [7,8,9]. PCR, qPCR, multiplex PCR, IHC, LAMP, and NGS are methods for early detection of pathogens, each with its own challenges and limitations, including specialized equipment, complexity, cross-reactivity, and advanced bioinformatics knowledge [6,9,10,11]. In instances of infestations by less virulent strains of ASFV or the long-term persistence of the virus in endemic areas, there is a significant demand for serological diagnosis and surveillance. The selection of a diagnostic method relies on factors such as the infection stage, resource availability, and specific diagnostic objectives. Considering these factors, ELISA seems to be the favored detection method, especially for conducting extensive tests in pig populations [1]. Recently, the World Organization for Animal Health (WOAH) acknowledged ELISA as the main serological method for ASF diagnosis [6,12]. This recognition has led to the development of various commercial kits based on ASFV proteins including ID. Vet (Montpellier, France), Ingenasa (Madrid, Spain), and Svanova (Uppsala, Sweden) [6,13]. Previous investigations have demonstrated that integrating multiple epitopes from antigenic proteins into the indirect ELISA approach offers several advantages. It enhances the identification of diverse antibody types in pigs, reducing the possibility of inaccurate negative or positive outcomes and allowing for a comprehensive assessment of ASFV exposure [14,15].

In response to the challenges posed by ASFV infection and the significant role played by various proteins at distinct infection stages, our study explores a novel approach in indirect ELISA design. Rather than focusing on a single protein target as seen in previous studies [16,17,18], we propose the use of multiple prominent B-cell epitopes named k3 derived from 11 ASFV antigenic proteins. This innovative approach aims to achieve heightened sensitivity and specificity compared to currently available commercial assays.

## 2. Materials and Methods

### 2.1. Standard Controls

ASFV positive (*n* = 1) and negative (*n* = 1) sera from the ASF Regional Laboratory of China (Lanzhou) were used as standard controls during this study. These samples were stored at the ASF Regional Laboratory, located at the Lanzhou Veterinary Research Institute, Chinese Academy of Agricultural Sciences (LVRI, CAAS).

### 2.2. Serum Samples Collection

For the current investigation, 90 ASFV-positive and 200 ASFV-negative samples were provided by the ASF Regional Laboratory of China (Lanzhou). Additionally, one reference positive serum each for PRRSV, CSFV, PCV2, and PRV was obtained from the China Veterinary Culture Collection Center, while positive serum (*n* = 1) for FMDV was sourced from the WOAH/National Foot-and-Mouth Diseases Reference Laboratory. Furthermore, clinical swine serum samples (*n* = 379) were provided by the ASF Regional Laboratory of China (Lanzhou), collected from adult pig farms in Gansu and Henan provinces spanning the years 2018 to 2022. All samples were carefully preserved at the ASF Regional Laboratory at the Lanzhou Veterinary Research Institute, Chinese Academy of Agricultural Sciences (LVRI, CAAS), for further analysis.

### 2.3. Epitopes Prediction

The FASTA sequences corresponding to the ASFV (ASFV-SY-18) proteins were obtained from the NCBI GenBank database (http://www.ncbi.nlm.nih.gov, accessed on 15 March 2023). A total 17 protein sequences were obtained including p72, pA104R, pB602L, p11.5, p12, p17, p14.5, p49, pE248R, p30, p54, pp62, pp220, pH240R, pK205R, p22, and pCD2v. In viral serum testing, B-cell epitopes play a crucial role in the immune response to viral infections and can be recognized by antibodies produced by B cells. Hence, two online epitope prediction tools ABCpred Prediction (http://crdd.osdd.net/raghava/abcpred/ABC_submission.html, accessed on 18 March 2023) and IEDB (http://www.iedb.org/, accessed on 18 March 2023) were utilized to identify the most immunogenic B-cell epitopes. Furthermore, the immunogenicity, hydrophilicity, and transmembrane region of the amino acid sequences of the proteins were assessed using the IEDB database.

### 2.4. Construction of the Multi-Epitope Protein k3

To assess the reactivity between the epitopes and serum samples to screen with ASFV positive and negative serum, we used the predicted epitopes that were synthesized by Solarbio (Beijing, China), and coated on ELISA plates (1 μg/mL). Then, the epitopes identified as dominant formed the foundation of the ASFV multi-epitope. These epitopes were connected using the “GGGGS” linker and supplemented with a 6 × His tag at the 3′ end to facilitate the protein purification. The designed sequence was purchased from Nanjing Genscript Company (Nanjing, China) and inserted into the pET-28a expression vector at BamH I and Xho I restriction enzyme sites. The vector constructs were verified by sequencing.

### 2.5. Expression of Multi-Epitope Protein k3 in E. coli

The plasmid recombinant k3 was transformed into *E. coli* BL21 (Invitrogen, Waltham, MA, USA) competent cells and cultured (220 rpm, 37 °C) in Luria broth medium containing kanamycin (50 μg/mL) for 12–14 h. Once the optical density (OD600) reached the range of 0.6 to 0.8, cellular induction was initiated by adding 1.0 mM isopropyl β-d-1-thiogalactopyranoside (IPTG; Solarbio, Beijing, China) and continued for 8 h at 37 °C. Boiling method was applied for both product supernatant and inclusion bodies as previously described [19]. The protein expressions were verified by Sodium dodecyl-sulfate polyacrylamide gel electrophoresis (SDS-PAGE).

### 2.6. Purification of Multi-Epitope Protein k3

Following optimization of the culture conditions, the *E. coli* cells were centrifuged (5000 rpm, 30 min, 4 °C), pellets obtained, and then re-suspended in pre-cold PBS on ice for ultrasonication. The lysate was centrifuged at 6682 rpm and 4 °C for 20 min and the recombinant k3 was purified using StrepTrap beads according to the manufacturer’s instructions (General Electric Company, Boston, MA, USA). The collected samples were identified by SDS–PAGE and transferred to polyvinylidene fluoride membrane (Millipore, Darmstadt, Germany). After a 2 h blocking step with 5% BSA, the target protein underwent probing with an anti-His tag polyclonal antibody for an additional 2 h. Protein bands were visualized using Clarity Western ECL substrate (Bio-Rad, Hercules, CA, USA) in conjunction with NcmECL Ultra (NCM Biotech Co., Ltd., Suzhou, China).

### 2.7. Establishment and Evaluation of Indirect ELISA of k3 Protein

#### 2.7.1. Matrix Titration

Ninety-six-well plates coated with recombinant protein at different concentrations (0.0625, 0.125, 0.25, 0.5, 1, and 2 µg/mL) diluted in carbonate buffer were incubated overnight at 4 °C following the procedure outlined in a previous study [20]. Briefly, washed three times with PBST containing 0.05% (*v*/*v*) Tween 20, the plates were blocked in 1% BSA in PBST for 1 h at 37 °C, and then rinsed one more time. ASFV positive and negative sera were diluted 1:100 in PBST containing 1% BSA and 2% goat serum, then 100 μL of diluted serum was added to each well and incubated for 1 h at 37 °C. The plates were washed three times, and 100 μL of diluted HRP-conjugated anti-pig antibody (1:20,000 in PBST containing 1% BSA and 2% goat serum) was added to each well, then incubated for 30 min at 37 °C. The plates were washed 3 times, and 100 μL of tetramethylbenzidine (TMB) was added and incubated for 10 to 15 min at 37 °C. Finally, 100 μL of 0.3 M H_2_SO_4_ was added to each well to determine the optical density (OD 450 nm), and the condition with the highest ratio of positive and negative sera (P/N value) was selected as the optimal working condition.

#### 2.7.2. Optimization of Experimental Parameters

Different blocking solutions (1% BSA, 2% BSA, 5% skim milk, 0.25% casein, and 5% Horse serum) were tested. In addition, several dilutions were applied for the ASFV sera (1:25, 1:50, 1:100, and 1:200) and HRP-conjugated anti-pig antibody (1:5000, 1:10,000, 1:20,000, and 1:40,000). Subsequently, the reaction times for the serum and HRP-conjugated anti-pig antibody were evaluated at 15, 30, 45, and 60 min.

#### 2.7.3. Determination of the Cutoff Value in k3-iELISA

To establish the cutoff value, 200 negative and 90 positive ASFV serum samples (ASF Regional Laboratory of China, Lanzhou) were tested by the k3-iELISA using the experimental optimal working conditions. The OD_450_ values of each serum sample were measured and interpreted using the formula X = ((Sample OD − Negative control OD)/(Positive control OD − Negative control OD)), as described previously [19]. The cutoff value was determined according to ROC curve analysis.

#### 2.7.4. Specificity and Sensitivity Tests

The developed ELISA method was employed to test positive sera of CSFV, PCV2, PRV, PRRSV, and FMDV including ASFV-positive and negative sera. To evaluate the sensitivity, ASFV-positive serum was diluted from 1:200 to 1:51,200 to determine the highest dilution of serum. Each sample was performed in two replicates and the mean average was calculated. In addition, both specificity and sensitivity were determined using the same formula ((Sample OD − Negative control OD)/(Positive control OD − Negative control OD)) as for the cutoff value analysis.

### 2.8. Detection of Clinical Samples and Comparison with the Commercial Kit

A total of 379 clinical swine serum samples were detected using the k3-iELISA developed in the current study.

Additionally, the samples were tested using a commercial ASFV antibody detection kit (Ingenasa, Madrid, Spain) provided by Qingdao RealVet Bio-Technology Co., Ltd., Qingdao, China. (ASF.K001/5) based on the p72 protein for comparison with the established ELISA method as previously described [1,16]. This comparison aimed to determine the effectiveness and coincidence rate, evaluating the consistency between the established ELISA and the commercial kit based on the test results of each sample.

### 2.9. Analyses

The epitopes were predicted using the ABCpred and IEDB websites, with selection based on the original sequence and a threshold value above 0.5.

For the screening of selected epitopes, matrix titrations, and method optimization, two replicates of each sample were performed. The ratio between the OD value of positive samples and the OD value of negative samples was obtained, as described elsewhere [17].

To determine the cutoff value, OD_450_ values of each serum sample were measured and interpreted using the formula = ((Sample OD − Negative control mean OD)/(Positive control mean OD − Negative control mean OD)) as described previously [18]. The PI value of each serum was analyzed by the ROC curve and Prism 9 software (GraphPad Software, La Jolla, CA, USA) to present the area under the curve (AUC) at a 95% CI.

For the method comparison with the commercial Kit, relative sensitivity, relative specificity, and coincidence rates were calculated as described previously [16].

Statistical significance was considered at *p* < 0.001.

## 3. Results

### 3.1. Screening of the Predominant Peptides

**Figure 1:**
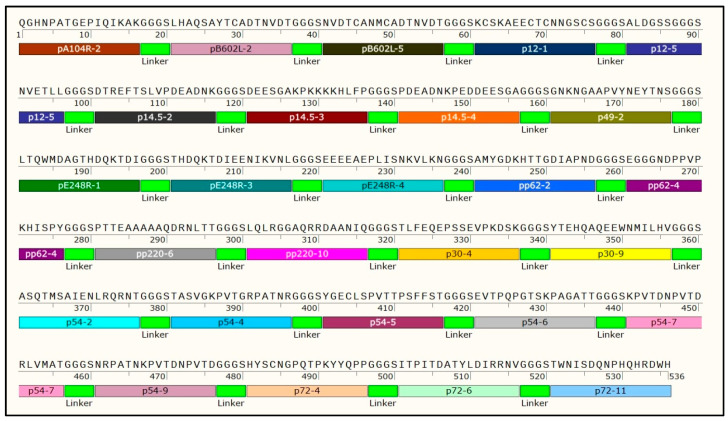
The illustration of k3 recombinant protein synthesis. The process of synthesizing the k3 recombinant protein by connecting optimized predominant peptides using GGGS linkers. 1–536 quantify the number of amino acids involved in the k3 synthesis.

Out of the 123 peptides predicted from 17 ASFV proteins, 27 were selected from 11 proteins as dominant linear epitopes (Table 1) for utilization in constructing the k3 multi-epitope fusion gene. The experiment involved coating ELISA plates with 123 peptides at a concentration of 1 μg/mL. Subsequently, the peptides were exposed to ASFV serum at a dilution of 1:50, followed by treatment with an HRP-conjugated anti-pig antibody at a dilution of 1:20,000. Predominant peptides were identified by evaluating the ratio of OD 450 nm values between the positive serum and negative serum (results shown in Supplementary Table S1). The coding sequence of multiple epitope k3 was then synthesized, incorporating the 27 selected dominant epitopes. As depicted in Figure 1, the illustration delineates the synthesis process of the k3 recombinant protein. This entails the connection of optimized predominant peptides utilizing GGGS linkers.

### 3.2. Expression, Purification, and Characterization of Recombinant Protein k3

**Figure 2:**
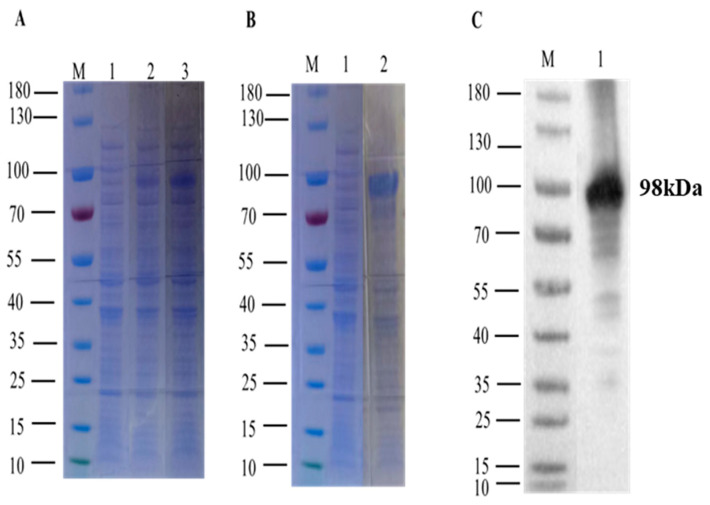
Expression and purification of the k3 protein: (**A**) M: Protein Marker, Lane 1: whole bacteria without induction, Lane 2: induced bacterial lysate 4 h, Lane 3: IPTG induced bacterial lysate 8 h (**B**) M: Protein Marker, Lane 1: Deposition of IPTG-induced cells, Lane 2: Supernatant of IPTG-induced cells (**C**) M: Protein Marker, Lane 1: Western blot analysis of k3 protein using anti-His tag.

The recombinant vector, leading to the expression and purification of the protein, was constructed. To distinguish it, the current identified protein with un-induced recombinant bacterial lysate SDS-PAGE analysis was used as indicated in (Figure 2A), which demonstrated the appearance of 98 kDa size post-IPTG induction. Furthermore, the k3 protein expressed was soluble, evident from its presence in the supernatant of the cell lysate (Figure 2B). The soluble protein fraction was purified and subsequently validated through Western blot analysis using a His-tag polyclonal antibody (Figure 2C).

### 3.3. Development of the k3-ELISA Method

**Figure 3:**
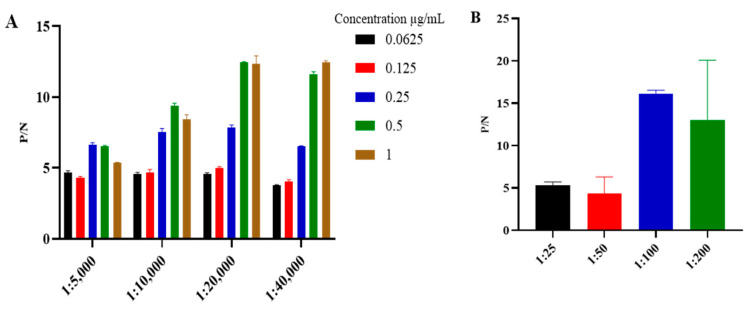
Determination of (**A**) antigen concentration and HRP-conjugated secondary antibody dilutions, and (**B**) ASFV serum dilutions.

**Figure 4:**
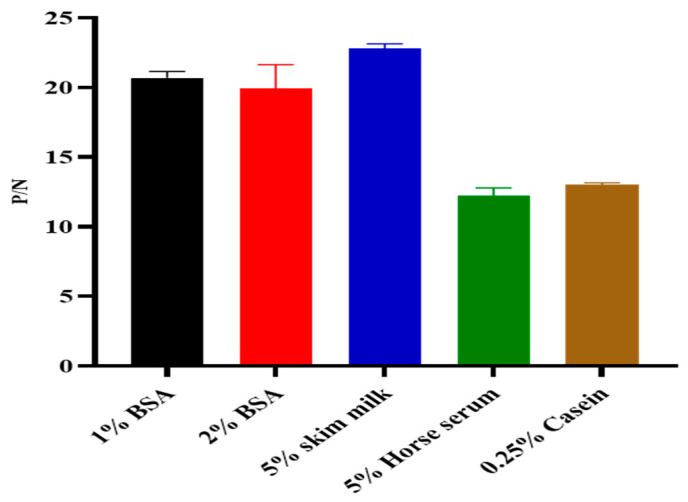
Optimization of determination of the appropriate blocking buffer.

**Figure 5:**
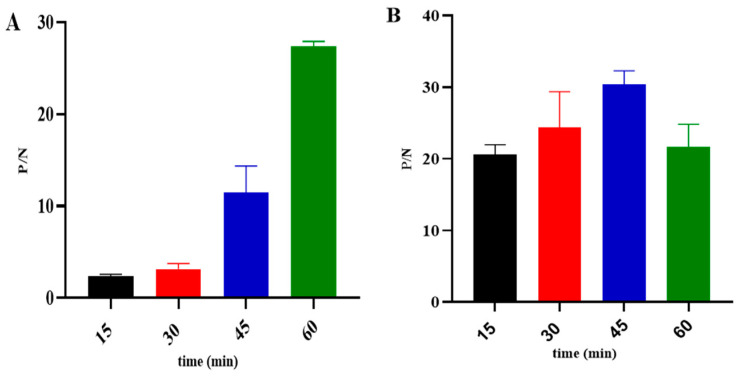
Optimization of ASFV serum incubation time (**A**) and HRP-conjugated secondary antibody incubation time (**B**).

The ideal concentrations and dilution factors of the k3 protein antigen, serum, and HRP-conjugated secondary antibody were determined using checkerboard titration. The results revealed an optimal antigen coating concentration of 0.5 µg/mL, along with optimal serum and HRP-conjugated secondary dilutions of 1:100 and 1:20,000, respectively (Figure 3A,B). Additionally, various optimization parameters were established. Specifically, a PBST solution containing 5% skim milk was identified as the optimal blocking solution (Figure 4). Furthermore, the incubation times for serum and HRP-conjugated secondary antibodies were assessed, with the most effective durations determined to be 60 min and 45 min at 37 °C, respectively (Figure 5A,B).

### 3.4. Cutoff Value Evaluation

**Figure 6:**
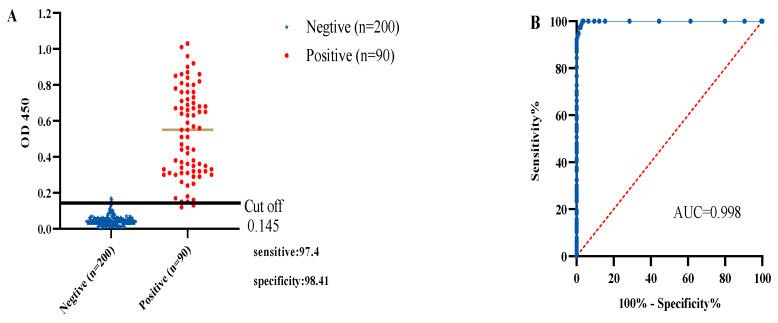
Determination of diagnostic sensitivity and specificity: (**A**) cutoff value and optimal diagnostic sensitivity and specificity (**B**) ROC curve showing accuracy value interpreted as the area under the curve (AUC = 0.998, *p* < 0.001).

The assay involved testing of 290 ASFV serum samples, consisting of 200 negative and 90 positive cases. Using the k3-iELISA method, a cutoff value of 0.145 (Figure 6A,B) was determined, marking samples as positive for values ≥ 0.145 and negative for values < 0.145. A ROC curve statistical analysis showed an Area Under the Curve (AUC) of 0.998 (*p* < 0.001). The method displayed a confidence level of 95.02%, signifying its high accuracy. This resulted in a dependable serological assay, boasting a diagnostic sensitivity of 97.4% and diagnostic specificity of 98.41%.

### 3.5. Assessment of Specificity and Sensitivity in k3-ELISA

**Figure 7:**
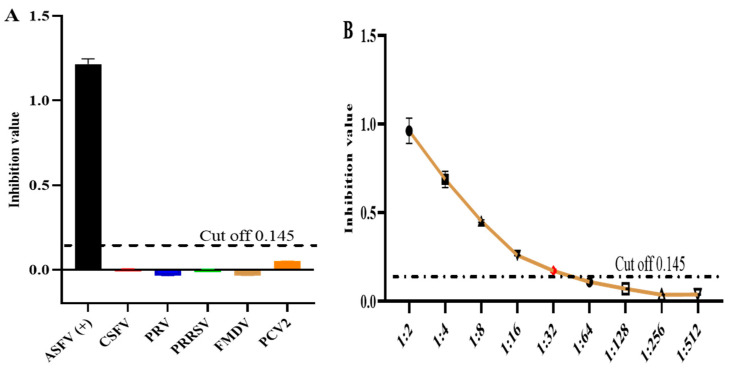
Specificity and sensitivity tests: (**A**) k3-iELISA detected no cross-reactions with sera-containing antibodies against five other porcine pathogens; (**B**) sensitivity test of the k3-iELISA.

To determine the specificity of the test, we tested serum harvested from pigs using the current method to detect positivity for antibodies against CSFV, PCV2, PRV, PRRSV, and FMDV. The present result revealed a negative result except for ASFV-positive serum. The current result of the established ELISA method has good specificity (Figure 7A). In addition, serial dilution was performed using the positive ASFV serum to test the sensitivity of the k3-ELISA with the results indicating that only the dilution factor of 1:32 gave a positive result according to the cutoff value (Figure 7B).

### 3.6. Comparison of the k3-ELISA Method to Commercial Kit

For the comparison analysis of the of the k3-iELISA with the commercial kit from INGENSA of Spain, we tested 379 clinical serums. The current finding regarding relative sensitivity, relative specificity, and coincidence rates, showed 100.00%, 98.00%, and 99.00%, respectively (Table 2).

## 4. Discussion

The rapid emergence of highly contagious diseases, such as ASFV, has necessitated the implementation of crucial diagnostic methods and the development of vaccines as essential measures for prevention and control [6]. Despite the global outbreak of ASF, there is currently no available vaccine specifically targeting this virus. Adding to the challenge, ASFV has exhibited multiple genotypes since its outbreak, contributing to the complexity of its immune mechanisms [20]. The discovery of B-cell epitopes associated with ASFV is critical to understanding virus–host interactions and plays a crucial role in the development of diagnostic tools and vaccines [21,22,23]. In this context, certain research efforts have proposed and implemented the use of different viral structural proteins to develop serologic diagnostic methods to detect the virus during its infection. The development of previous ELISA kits targeting a limited number of epitopes from fewer proteins might not fully capture the complexity of ASFV infection dynamics, as multiple proteins are known to play crucial roles at different stages of the infection process. In this study, we aimed to address this limitation by formulating a panel of B-cell epitopes derived from various ASFV proteins (p72, pA104R, pB602L, p11.5, p12, p17, p14.5, p49, pE248R, p30, p54, pp62, pp220, pH240R, pK205R, p22, and pCD2v) that exhibit robust antigenicity and induce increased antibody titers in infected pigs [24,25], followed by the construction of a recombinant protein designated k3. This k3 protein was expressed in *E. coli*, and its utility as an antigen was investigated for diagnostic purposes in a novel iELISA for the serologic diagnosis of ASF infection.

There are various diagnostic methods necessary for the detection of the viral DNA, such as quantitative real-time PCR and in situ hybridization with nucleic acid probes, and commercial ELISA kits developed for the detection of ASFV antibodies focusing predominantly on key proteins, including p30, p54, and p72 [26,27]. Serologic diagnosis provides a stable detection method, excellent specificity, high sensitivity and ease of use, and is cost-effective. ELISA as antibody detection technology plays an important role in ASFV epidemic diagnosis. Due to these advantages, ELISA is well suited for the detection of large numbers of samples and is therefore a widely used and practical method in various fields during disease surveillance and control. The reliability, ease of use, and cost-effectiveness of ELISA contribute to its popularity and extensive application in both research and diagnostic settings [27,28,29].

In the present study, we predicted, synthesized, and screened B-cell epitopes from 11 ASFV proteins. From these proteins, we selected 27 predominant epitopes and constructed a recombinant protein named k3, which was expressed in *E. coli* competent cells, purified, and utilized as an antigen for ELISA method development. The developed method was then validated using a commercial kit.

The specificity test showed that there was no cross-reactivity with antibodies derived from other pathogens, including PRRSV, PCV2, CSFV, PRV, and FMDV of swine. Moreover, the sensitivity test of the developed ELISA was evaluated in the present assessment and the result showed that the current ELISA kit was capable of detecting a commercially positive ASFV serum at a high dilution of 1:32, which is consistent with the results of a previous study [17]. In addition, the agreement rate between the k3-iELISA and INGENASA’s commercial ASFV antibody detection kit was 99.00% [14,16]. These results emphasize the significant clinical potential of the k3-iELISA. The efficacy of the k3-indirect ELISA lies in its ability to detect ASFV infection in affected animals at early, intermediate, and late stages. This is attributed to the comprehensive role played by all proteins involved in the construction of the k3 recombinant protein, setting it apart from existing methods [14,16,17]. The assay shows its capability to identify ASFV-specific antibodies in samples at different stages of infection, providing a valuable tool and accurate detection of ASFV.

## 5. Conclusions

Conclusively, our study successfully screened ASFV B-cell epitopes and constructed a recombinant protein k3, which was purified from *E. coli* cells. The newly established indirect enzyme-linked immunosorbent assay (iELISA) using k3 as the coated antigen demonstrated good sensitivity and specificity in detecting ASFV antibodies. Additionally, the k3-iELISA was demonstrated to be a reliable method, establishing a solid groundwork for future epidemiological studies.

## Figures and Tables

**Table 1:** List of dominant linear epitope sequences selected.

No	Epitope Name	Protein Name	Sequence of Dominant Linear Epitope	Position	Score
1	pA104R-2	pA104R	QGHNPATGEPIQIKAK	70–85	0.85
2	pB602L-2	pB602L	LHAQSAYTCADTNVDT	20–35	0.94
3	pB602L-5	pB602L	NVDTCASMCADTNVDT	32–47	0.91
4	p12-1	p12	KCSKAEECTCNNGSCS	42–57	0.81
5	p12-5	p12	ALDGSSGGGSNVETLL	2–17	0.60
6	p14.5-2	p14.5	DTREFTSLVPDEADNK	80–95	0.91
7	p14.5-3	p14.5	DEESGAKPKKKKHLFP	99–114	0.89
8	p14.5-4	p14.5	PDEADNKPEDDEESGA	99–114	0.83
9	p49-2	p49	GNKNGAAPVYNEYTNS	41–56	0.93
10	pE248R-1	pE248R	LTQWMDAGTHDQKTDI	91–106	0.94
11	pE248R-3	pE248R	THDQKTDIEENIKVNL	99–114	0.89
12	pE248R-4	pE248R	EEEEAEPLISNKVLKN	229–244	0.85
13	p72-4	p72	HYSCNGPQTPKYYQPP	311–326	0.95
14	p72-6	p72	ITPITDATYLDIRRNV	295–310	0.94
15	p72-11	p72	TWNISDQNPHQHRDWH	473–488	0.9
16	p30-4	p30	TLFEQEPSSEVPKDSK	121–136	0.82
17	p30-9	p30	YTEHQAQEEWNMILHV	74–89	0.77
18	p54-2	p54	ASQTMSAIENLRQRNT	159–174	0.87
19	p54-4	p54	TASVGKPVTGRPATNR	95–110	0.85
20	p54-5	p54	YGECLSPVTTPSFFST	14–29	0.83
21	p54-6	p54	EVTPQPGTSKPAGATT	80–95	0.82
22	p54-7	p54	KPVTDNPVTDRLVMAT	115–130	0.81
23	p54-9	p54	NRPATNKPVTDNPVTD	109–124	0.8
24	pp62-2	pp62	AMYGDKHTTGDIAPND	422–437	0.89
25	pp62-4	pp62	EGGGNDPPVPKHISPY	156–171	0.88
26	pp220-6	pp220	PTTEAAAAAQDRNLTT	54–69	0.84
27	pp220-10	pp220	LQLRGGAQRRDAANIQ	233–248	0.81

**Table 2:** Comparison of k3-indirect ELISA and the commercial kits.

		INGENASA-Indirect ELISA		Total
		Positive	Negative	
k3-iELISA	positive	145	4	149
	negative	0	230	230
	Total	145	234	379

Relative sensitivity = 100.00% (145/145), relative specificity = 98.00% (230/234), coincidence rate = 99.00% (375/379).

## Data Availability

The FASTA sequences corresponding to the ASFV proteins were obtained from the NCBI GenBank database (http://www.ncbi.nlm.nih.gov, accessed on 15 March 2023). Further information is available from the corresponding author.

## Supplementary Materials

The following supporting information can be downloaded at https://www.mdpi.com/article/10.3390/microorganisms12050943/s1, Table S1. Epitopes screening. The table depicts the P/N (Positive/Negative) values obtained from the evaluation of 27 selected epitopes by ELISA.
