# Supplementary material for: Establishment of an ELISA Based on a Recombinant Antigenic Protein Containing Multiple Prominent Epitopes for Detection of African Swine Fever Virus Antibodies
Source: Microorganisms. 2024 May 7;12(5):943. doi: 10.3390/microorganisms12050943 (PMC11124277; doi:10.3390/microorganisms12050943)
Supplement: Supplementary file 1 [file microorganisms-12-00943-s001.zip › microorganisms-2982137-supplementary.pdf]

	A104R-2	B602L-2	B602L-5	P12-1	P12-5	p14.5-2	p14.5-3	p14.5-4	P49-2
P	0.7452	1.0983	1.1455	1.3287	1.1676	1.8634	1.0542	1.1657	1.3181
	0.7565	1.1053	1.1474	1.336	1.1846	1.891	1.0998	1.1336	1.2383
	0.75085	1.1018	1.14645	1.33235	1.1761	1.8772	1.077	1.14965	1.2782
N	0.3805	0.4565	0.6794	0.5589	0.4486	0.6266	0.341	0.4474	0.631
	0.3183	0.4673	0.6171	0.5324	0.4123	0.6685	0.3603	0.4371	0.6367
	0.3494	0.4619	0.64825	0.54565	0.43045	0.64755	0.35065	0.44225	0.63385
P/N	2.14897	2.385365	1.768531	2.441767	2.732257	2.898927	3.071439	2.599548	2.016565

Table S1. Epitopes screening. The table depicts the P/N (Positive/Negative) values obtained from the evaluation of 27 selected epitopes by ELISA.

Screening of epitopes

pE248R-1	pE248R-3	pE248R-4	P72-4	P72-6	P72-11	P30-4	P30-9	P54-2	P54-4
1.3161	1.3259	2.3885	1.2624	1.3261	1.4015	1.3282	1.5744	2.4357	1.401
1.3152	1.3888	2.4097	1.2589	1.4583	1.3921	1.3342	1.5882	2.3871	1.4223
1.31565	1.35735	2.3991	1.26065	1.3922	1.3968	1.3312	1.5813	2.4114	1.41165
0.6652	0.6609	0.6625	0.6514	0.7335	0.7436	0.7259	0.749	0.3349	0.2805
0.6407	0.6749	0.6595	0.6488	0.7205	0.741	0.6978	0.7211	0.3222	0.2789
0.65295	0.6679	0.661	0.6501	0.727	0.7423	0.71185	0.73505	0.32855	0.2797
2.014932	2.032265	3.629501	1.939163	1.914993	1.881719	1.870057	2.151282	7.339522	5.047015

P54-5	P54-6	P54-7	P54-9	pp62-2	pp62-4	pp220-6	pp220-10
3.0908	0.5595	0.7614	1.0174	1.5088	1.3044	1.5432	1.4388
3.1274	0.6251	0.7835		1.5051	1.307	1.5326	1.4358
3.1091	0.5923	0.77245	1.0174	1.50695	1.3057	1.5379	1.4373
0.2651	0.2618	0.252	0.2187	0.6251	0.6695	0.5188	0.6031
0.2645	0.2544	0.2561	0.2188	0.6208	0.6036	0.5204	0.5924
0.2648	0.2581	0.25405	0.21875	0.62295	0.63655	0.5196	0.59775
11.74131	2.294847	3.040543	4.650971	2.419054	2.051214	2.959777	2.404517
